# Comparative Evaluation of the Effectiveness of Nivolumab Versus Axitinib in the Second-Line Treatment of Patients with Metastatic Renal Cell Carcinoma: A Two-Center Experience

**DOI:** 10.3390/jcm15187146

**Published:** 2026-09-15

**Authors:** İrfan Buğday, İlknur Deliktaş Onur, Mevlüde İnanç, Metin Özkan, Oktay Bozkurt

**Affiliations:** 1 Medical Oncology Department, Kayseri City Hospital, 38070 Kayseri, Türkiye; 2 Medical Oncology Department, Dr. Abdurrahman Yurtaslan Ankara Oncology Hospital, 06200 Ankara, Türkiye; 3 Medical Oncology Department, Erciyes University Medical School, 38280 Kayseri, Türkiye

**Keywords:** axitinib, nivolumab, metastatic renal cell carcinoma, second-line treatment, progression-free survival, overall survival

## Abstract

**Objectives:** This study aimed to compare the effectiveness of nivolumab and axitinib as second-line treatments in patients with metastatic renal cell carcinoma (mRCC) who had progressed after one prior tyrosine kinase inhibitor (TKI)-based therapy. **Methods**: We retrospectively evaluated 68 patients with mRCC who received nivolumab or axitinib as second-line treatment at two centers. Progression-free survival (PFS) and overall survival (OS) were estimated using the Kaplan–Meier method and compared using the log-rank test. Multivariable Cox proportional hazards regression models were performed to evaluate the association of treatment with PFS and OS after adjustment for International Metastatic Renal Cell Carcinoma Database Consortium (IMDC) risk group and year of second-line treatment initiation. **Results**: Nineteen patients received nivolumab, and 49 received axitinib. Median PFS was 9.27 months (95% confidence interval [CI], 5.75–12.79) with nivolumab and 5.87 months (95% CI, 4.73–7.01) with axitinib (log-rank *p* = 0.011). Median OS was 48.27 months (95% CI, 4.16–92.37) and 30.20 months (95% CI, 19.95–40.44), respectively (log-rank *p* = 0.027). In multivariable analysis, axitinib was associated with a significantly higher risk of progression or death compared with nivolumab (hazard ratio [HR], 3.494; 95% CI, 1.690–7.225; *p* = 0.001). IMDC risk group was also significantly associated with PFS (overall *p* = 0.003), whereas year of second-line treatment initiation was not (HR, 1.070; 95% CI, 0.883–1.296; *p* = 0.489). For OS, axitinib was associated with a significantly higher risk of death compared with nivolumab (HR, 2.410; 95% CI, 1.200–4.838; *p* = 0.013), while neither IMDC risk group (overall *p* = 0.477) nor treatment year (HR, 1.102; 95% CI, 0.922–1.317; *p* = 0.286) was significantly associated with OS. **Conclusions**: In this two-center retrospective cohort, nivolumab was associated with longer PFS and OS than axitinib in the second-line treatment of mRCC, and these associations remained significant after adjustment for IMDC risk group and year of treatment initiation. However, the retrospective design, small sample size, imbalance in treatment allocation and nephrectomy rates, and potential residual confounding limit causal interpretation. Prospective studies are warranted to confirm these findings.

## 1. Introduction

More than 80% of kidney cancers originate in the renal parenchyma, while the remainder arise from the renal pelvis [1]. Localized renal cell carcinoma (RCC) is primarily treated with partial or radical nephrectomy [2], with a 5-year relative survival rate of approximately 94% [3]. For patients with regional and distant disease, the corresponding 5-year relative survival rates are approximately 78% and 20%, respectively [3].

Historically, cytokine-based therapies such as interferon-alpha and high-dose interleukin-2 were used in the treatment of metastatic RCC, although their efficacy was limited [4,5,6]. The identification of von Hippel–Lindau gene alterations and their role in vascular endothelial growth factor (VEGF) signaling led to the development of VEGF-targeted therapies [7]. Tyrosine kinase inhibitors (TKIs), including sunitinib and pazopanib, subsequently became established treatment options for advanced RCC [8,9].

The development of immune checkpoint inhibitors (ICIs) has further transformed the treatment landscape of metastatic RCC. Programmed death ligand-1 (PD-L1) expression and the tumor immune microenvironment have been implicated in RCC biology and prognosis [10,11,12]. Early clinical studies demonstrated the activity of programmed death-1 (PD-1) blockade in advanced solid tumors, including RCC [13]. In the phase III CheckMate 025 trial, nivolumab significantly improved overall survival compared with everolimus in previously treated advanced RCC, establishing nivolumab as an important second-line treatment option [14]. Axitinib has also demonstrated efficacy in previously treated metastatic RCC and remains an established targeted therapy option in this setting [15].

The treatment landscape of metastatic RCC has continued to evolve, and immune checkpoint inhibitor-based combinations are now widely used in the first-line setting [2]. However, treatment selection after progression following prior TKI-based therapy remains clinically relevant, particularly in patients who were initially treated with targeted therapy.

The International Metastatic Renal Cell Carcinoma Database Consortium (IMDC) risk classification is an established prognostic model for patients with metastatic RCC and incorporates clinical and laboratory parameters to stratify patients according to prognosis [16,17]. However, real-world data directly comparing nivolumab and axitinib as second-line treatment remain limited, particularly in routine clinical practice.

Therefore, this study aimed to compare the effectiveness of nivolumab and axitinib as second-line treatments in patients with metastatic RCC who had progressed after one prior TKI-based therapy. We also evaluated the prognostic significance of IMDC risk group and year of second-line treatment initiation in relation to progression-free survival (PFS) and overall survival (OS).

## 2. Materials and Methods

### 2.1. Patients

This retrospective, two-center study included patients with metastatic renal cell carcinoma (mRCC) who received nivolumab or axitinib as second-line treatment after progression following one prior tyrosine kinase inhibitor (TKI)-based therapy. Patients were treated at the Department of Medical Oncology, Erciyes University, and the University of Health Sciences Dr. Abdurrahman Yurtaslan Ankara Oncology Training and Research Hospital between 1 January 2012 and 30 September 2022. Patients with incomplete clinical or survival data were excluded from the analysis.

The study was approved by the Erciyes University Clinical Research Ethics Committee on 6 September 2023 (decision no. 580). Because of the retrospective nature of the study, the requirement for informed consent was waived in accordance with applicable national regulations.

### 2.2. Data Collection

Clinical and demographic data were retrospectively obtained from electronic medical records and institutional databases. The following variables were recorded: age, sex, Eastern Cooperative Oncology Group performance status (ECOG PS), nephrectomy status, histological subtype, first-line TKI treatment, metastatic sites, IMDC risk group, year of second-line treatment initiation, second-line treatment type, treatment response, and adverse events.

Adverse events documented during second-line treatment were also recorded and are presented in Table 1. Treatment response was assessed according to the Response Evaluation Criteria in Solid Tumors version 1.1 (RECIST 1.1), and the best overall response during second-line treatment was recorded.

The data cutoff date was 31 August 2023.

### 2.3. Treatment and Outcome Definitions

Patients received either nivolumab or axitinib as second-line treatment following progression on one prior TKI-based therapy. Nivolumab was administered according to standard clinical practice, and axitinib was administered at the discretion of the treating physician.

Progression-free survival (PFS) was defined as the time from initiation of second-line treatment to radiological or clinical disease progression or death from any cause, whichever occurred first. Patients without progression or death at the date of last follow-up were censored at that time.

Overall survival (OS) was defined as the time from initiation of second-line treatment to death from any cause. Patients who were alive at the last follow-up were censored at that date.

The IMDC risk classification was categorized as favorable, intermediate, or poor risk according to established criteria. The year of second-line treatment initiation was analyzed as a continuous variable in the multivariable models.

### 2.4. Statistical Analysis

Statistical analyses were performed using IBM SPSS Statistics version 25.0 (IBM Corp., Armonk, NY, USA). Continuous variables were summarized using means and standard deviations or medians and ranges, as appropriate. Categorical variables were presented as frequencies and percentages. Comparisons between treatment groups were performed using the chi-square test or Fisher’s exact test for categorical variables and the independent-samples *t*-test or Mann–Whitney U test for continuous variables, as appropriate.

PFS and OS were estimated using the Kaplan–Meier method and compared between treatment groups using the log-rank test. Cox proportional hazards regression models were used to evaluate the associations of second-line treatment, IMDC risk group, and year of second-line treatment initiation with PFS and OS. Hazard ratios (HRs) with 95% confidence intervals (CIs) were reported. The proportional hazards assumption was assessed visually using log-minus-log survival plots. The approximately parallel curves supported the adequacy of the proportional hazards assumption for the treatment comparison.

All statistical tests were two-sided, and a *p*-value < 0.05 was considered statistically significant.

## 3. Results

### 3.1. Patient Characteristics

A total of 68 patients with metastatic renal cell carcinoma (mRCC) were included in the study. Nineteen patients (27.9%) received nivolumab, and 49 (72.1%) received axitinib as second-line treatment. The median age of the study population was 57 years (range, 26–83 years), and 44 patients (64.7%) were male. The median year of second-line treatment initiation was 2020 in both treatment groups, with no statistically significant difference between the groups (*p* = 0.158).

Overall, 52 patients (76.5%) had undergone nephrectomy. Nephrectomy was performed in 9 of 19 patients (47.4%) in the nivolumab group and 34 of 49 patients (69.4%) in the axitinib group. Other demographic and clinical characteristics of the patients according to second-line treatment are presented in Table 1.

The median follow-up duration was 27.63 months (range, 0.60–73.07 months). At the end of follow-up, 57 patients (83.8%) had died, and 11 (16.2%) were censored. In the nivolumab group, 12 patients (63.2%) had died, and 7 (36.8%) were censored, whereas in the axitinib group, 45 patients (91.8%) had died and 4 (8.2%) were censored.

### 3.2. Progression-Free Survival

**Figure 1:**
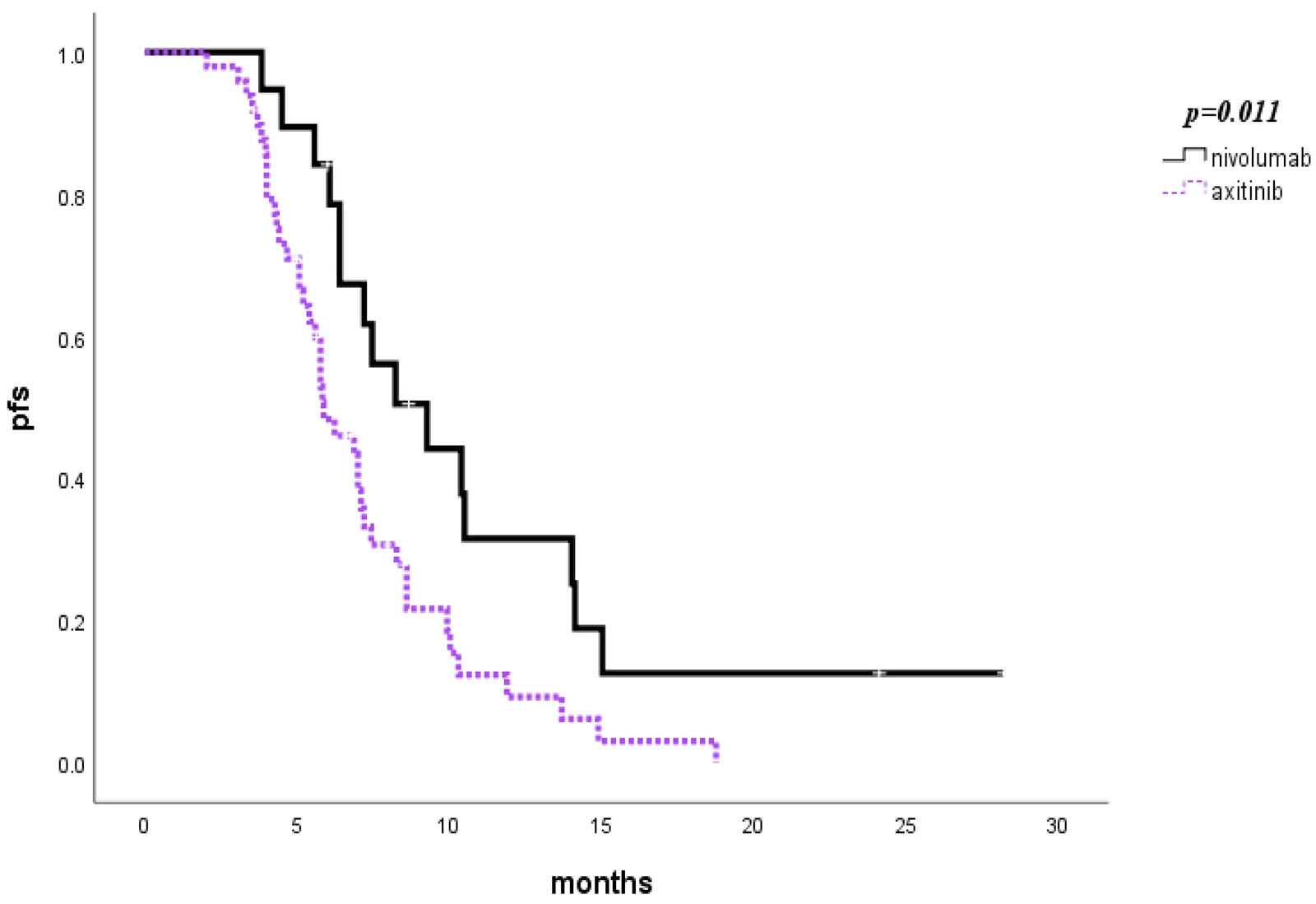
Progression-free survival according to second-line treatment with nivolumab or axitinib.

The median PFS was 9.27 months (95% CI, 5.75–12.79) in the nivolumab group and 5.87 months (95% CI, 4.73–7.01) in the axitinib group. The difference between the treatment groups was statistically significant (log-rank *p* = 0.011) (Figure 1).

Progression or death occurred in 15 of 19 patients (78.9%) in the nivolumab group and 41 of 49 patients (83.7%) in the axitinib group. Four patients (21.1%) in the nivolumab group and 8 patients (16.3%) in the axitinib group were censored for PFS.

In the multivariable Cox proportional hazards analysis adjusted for second-line treatment, IMDC risk group, and year of second-line treatment initiation, axitinib was associated with a significantly higher risk of progression or death compared with nivolumab (HR, 3.494; 95% CI, 1.690–7.225; *p* = 0.001). IMDC risk group was significantly associated with overall PFS (*p* = 0.003). Compared with favorable-risk disease, intermediate-risk disease was associated with a higher risk of progression or death (HR, 3.146; 95% CI, 1.570–6.302; *p* = 0.001), whereas poor-risk disease was not significantly associated with PFS (HR, 1.533; 95% CI, 0.467–5.035; *p* = 0.482). Year of second-line treatment initiation was not significantly associated with PFS (HR, 1.070; 95% CI, 0.883–1.296; *p* = 0.489) (Table 2).

### 3.3. Overall Survival

**Figure 2:**
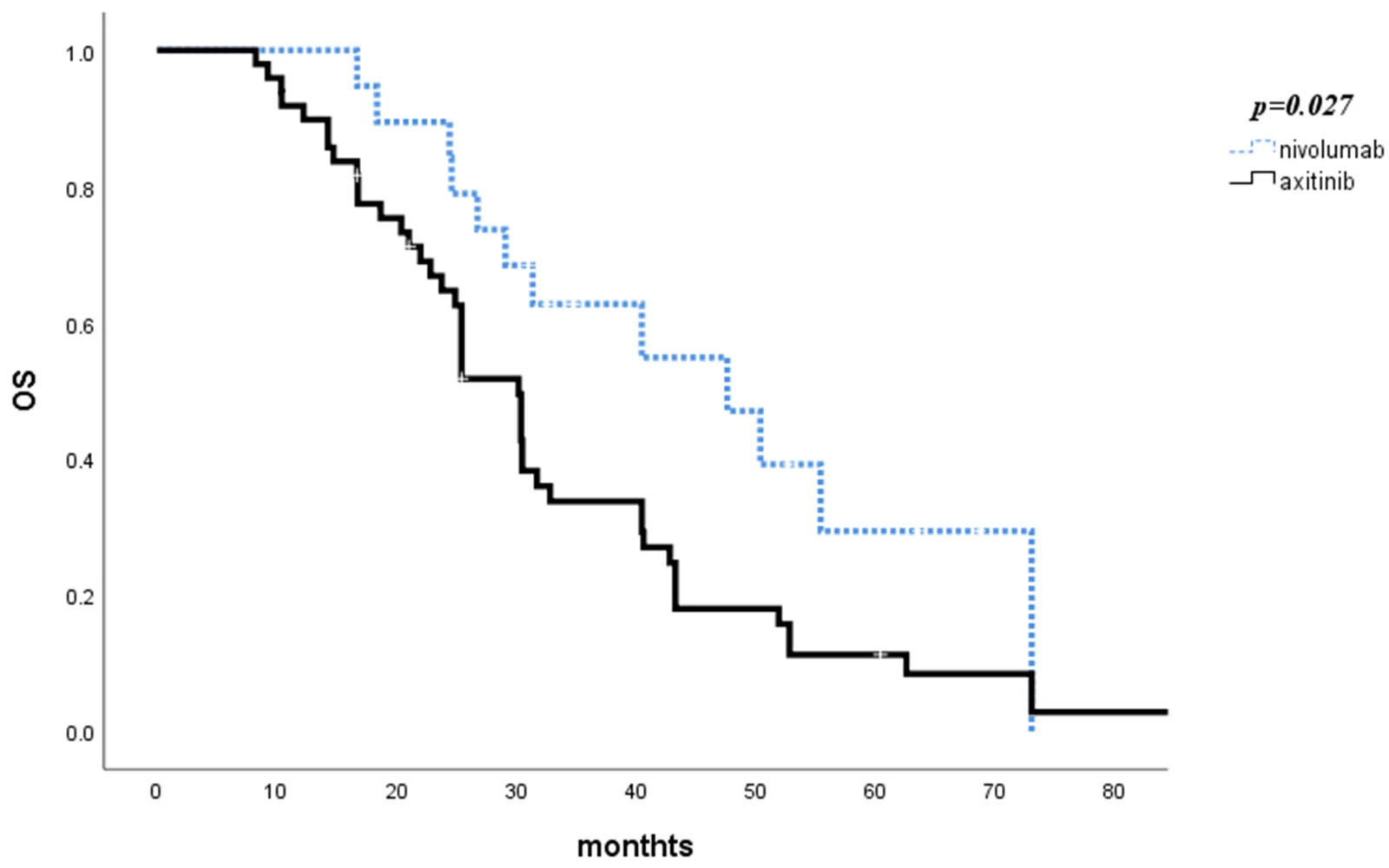
Overall survival according to second-line treatment with nivolumab or axitinib.

The median OS was 48.27 months (95% CI, 4.16–92.37) in the nivolumab group and 30.20 months (95% CI, 19.95–40.44) in the axitinib group. The difference between the treatment groups was statistically significant (log-rank *p* = 0.027) (Figure 2).

Twelve of 19 patients (63.2%) in the nivolumab group and 45 of 49 patients (91.8%) in the axitinib group died during follow-up. Seven patients (36.8%) in the nivolumab group and 4 patients (8.2%) in the axitinib group were censored.

In the multivariable Cox proportional hazards analysis adjusted for second-line treatment, IMDC risk group, and year of second-line treatment initiation, axitinib was associated with a significantly higher risk of death compared with nivolumab (HR, 2.410; 95% CI, 1.200–4.838; *p* = 0.013). IMDC risk group was not significantly associated with OS overall (*p* = 0.477). Compared with favorable-risk disease, neither intermediate-risk disease (HR, 1.703; 95% CI, 0.664–4.366; *p* = 0.268) nor poor-risk disease (HR, 1.035; 95% CI, 0.544–1.971; *p* = 0.915) was significantly associated with OS. Year of second-line treatment initiation was also not significantly associated with OS (HR, 1.102; 95% CI, 0.922–1.317; *p* = 0.286) (Table 2).

### 3.4. Treatment Response

Treatment response was evaluated according to the Response Evaluation Criteria in Solid Tumors version 1.1 (RECIST 1.1), and the best overall response during second-line treatment was recorded. Complete response was observed in 2 patients (10.5%) in the nivolumab group and in none of the patients in the axitinib group. Partial response was observed in 9 (47.4%) and 12 (24.5%) patients, respectively, while stable disease was observed in 5 (26.3%) and 25 (51.0%) patients. Progressive disease occurred in 3 (15.8%) patients receiving nivolumab and 12 (24.5%) receiving axitinib. The distribution of best overall response categories differed significantly between the treatment groups (Fisher’s exact *p* = 0.025).

## 4. Discussion

In this two-center retrospective study of patients with metastatic renal cell carcinoma who received second-line treatment after progression on a prior TKI-based therapy, nivolumab was associated with significantly longer PFS and OS than axitinib. The median PFS was 9.27 months with nivolumab versus 5.87 months with axitinib, and the median OS was 48.27 versus 30.20 months, respectively. Importantly, these associations remained statistically significant after adjustment for IMDC risk group and year of second-line treatment initiation, with axitinib associated with a higher risk of progression or death (HR, 3.494) and death (HR, 2.410) compared with nivolumab.

The longer OS observed with nivolumab is broadly consistent with the results of the phase III CheckMate 025 trial, which demonstrated improved overall survival with nivolumab compared with everolimus in patients with previously treated advanced RCC [14]. Although the AXIS trial established axitinib as an effective second-line treatment option compared with sorafenib [15], direct comparisons between nivolumab and axitinib in routine clinical practice remain limited. Our findings therefore provide additional real-world data regarding the comparative effectiveness of these two treatment approaches.

The treatment era was considered an important potential source of confounding because nivolumab became available for previously treated advanced RCC later than axitinib. In the present cohort, the median year of second-line treatment initiation was 2020 in both treatment groups, with no statistically significant difference between groups (*p* = 0.158). Furthermore, year of treatment initiation was included as a continuous covariate in the multivariable Cox models and was not significantly associated with either PFS (HR, 1.070; 95% CI, 0.883–1.296; *p* = 0.489) or OS (HR, 1.102; 95% CI, 0.922–1.317; *p* = 0.286). These findings do not suggest that treatment era alone explained the observed differences in outcomes, although residual confounding related to changes in treatment selection and clinical practice over time cannot be excluded.

IMDC risk group was significantly associated with PFS in the multivariable analysis, particularly for patients with intermediate-risk disease compared with those with favorable-risk disease. In contrast, IMDC risk group was not significantly associated with OS. This difference may partly reflect the relatively small sample size and the number of events within individual risk categories. Nevertheless, the inclusion of the IMDC risk group in the multivariable models allowed adjustment for an established prognostic factor in metastatic RCC [16,17].

Another potential source of confounding was the imbalance in nephrectomy status between treatment groups. Nephrectomy was performed in 47.4% of patients receiving nivolumab and 69.4% of those receiving axitinib. Because nephrectomy status may be associated with disease burden, patient selection, and prognosis, this imbalance could have influenced the observed treatment effect. Although nephrectomy was not included in the final multivariable models, its imbalance between treatment groups should be considered when interpreting the results. Cytoreductive nephrectomy remains an important component of the historical management of metastatic RCC, although its role has evolved with the introduction of modern systemic therapies [18].

The first-line treatment landscape of metastatic RCC has also changed substantially over time, with immune checkpoint inhibitor-based combinations now widely used in the first-line setting [2]. However, the present study included patients who had received a prior TKI-based treatment, reflecting an earlier treatment pathway that remains relevant for patients treated during the study period and for those who progress after targeted therapy.

This study has several limitations. First, its retrospective design and relatively small sample size limit the ability to establish a causal relationship between treatment choice and survival outcomes. Second, treatment allocation was not randomized, and baseline differences between the treatment groups, particularly nephrectomy status, may have resulted in residual confounding. Third, the study included patients treated over several years during a period of substantial changes in the management of metastatic RCC, and unmeasured differences in treatment selection and supportive care may have influenced outcomes. Fourth, the relatively small number of patients and events limited the ability to perform more extensive adjusted analyses or subgroup analyses. The relatively small number of patients and the distribution of treatment years may also have introduced uncertainty into the estimation of calendar-year effects. Therefore, the treatment-year estimate should be interpreted cautiously and should not be considered evidence of a true year-to-year biological effect on survival. Finally, the study was conducted at two centers, which may limit the generalizability of the findings.

Despite these limitations, the study provides real-world comparative data on nivolumab and axitinib as second-line treatment options in patients with metastatic RCC after prior TKI therapy. The consistency of the treatment association across unadjusted and adjusted analyses suggests that the observed difference may be clinically relevant, while the observational nature of the study warrants cautious interpretation.

## 5. Conclusions

In this two-center retrospective study, nivolumab was associated with significantly longer PFS and OS compared with axitinib in patients with metastatic RCC receiving second-line treatment after prior TKI therapy. These associations remained statistically significant after adjustment for IMDC risk group and year of second-line treatment initiation. However, the retrospective design, small sample size, imbalance in treatment allocation and nephrectomy rates, changes in treatment patterns over time, and potential residual confounding limit causal interpretation. Prospective studies are warranted to confirm these findings.

## Figures and Tables

**Table 1:** Baseline demographic and clinical characteristics and adverse events according to second-line treatment.

Characteristic	Nivolumab (*n* = 19)	Axitinib (*n* = 49)	*p*-Value
**Age, years, mean ± SD**	58.21 ± 9.40	53.24 ± 11.48	0.098
**Sex, *n* (%)**			0.121 *
Male	17 (89.5)	34 (69.4)	
Female	2 (10.5)	15 (30.6)	
**ECOG performance status, *n* (%)**			0.763 *
0	5 (26.3)	12 (24.5)	
1	13 (68.4)	36 (73.5)	
2	1 (5.3)	1 (2.0)	
**Histological type, *n* (%)**			0.439 *
Clear cell	16 (84.2)	41 (83.7)	
Papillary	2 (10.5)	5 (10.2)	
Sarcomatoid	0 (0.0)	3 (6.1)	
Unclassified	1 (5.3)	0 (0.0)	
**First-line TKI, *n* (%)**			0.111
Sunitinib	6 (31.6)	26 (53.1)	
Pazopanib	13 (68.4)	23 (46.9)	
**Previous nephrectomy, *n* (%)**			0.091
No	10 (52.6)	15 (30.6)	
Yes	9 (47.4)	34 (69.4)	
**Previous chemoembolization, *n* (%)**			1.000 *
No	18 (94.7)	45 (91.8)	
Yes	1 (5.3)	4 (8.2)	
**IMDC risk group, *n* (%)**			0.724 *
Favorable	3 (15.8)	13 (26.5)	
Intermediate (1–2)	13 (68.4)	30 (61.2)	
Poor (≥3)	3 (15.8)	6 (12.2)	
**Year of second-line treatment initiation, median (IQR)**	2020 (2)	2020 (3)	0.158 †
**Lung metastasis, *n* (%)**			0.338 *
No	3 (15.8)	3 (6.1)	
Yes	16 (84.2)	46 (93.9)	
**Bone metastasis, *n* (%)**			0.069
No	4 (21.1)	22 (44.9)	
Yes	15 (78.9)	27 (55.1)	
**Liver metastasis, *n* (%)**			0.415
No	14 (73.7)	31 (63.3)	
Yes	5 (26.3)	18 (36.7)	
**Brain metastasis, *n* (%)**			1.000 *
No	18 (94.7)	46 (93.9)	
Yes	1 (5.3)	3 (6.1)	
**Adverse events during second-line treatment, *n* (%)**			
Fatigue	5 (26.3)	20 (40.8)	0.401 *
Appetite loss	4 (21.1)	16 (32.7)	0.393 *
Oral mucositis	3 (15.8)	7 (14.3)	1.000 *
Diarrhea	3 (15.8)	9 (18.4)	1.000 *
Hypertension	4 (21.1)	16 (32.7)	0.393 *
Hypothyroidism	7 (36.8)	13 (26.5)	0.554 *
Thrombocytopenia	1 (5.3)	3 (6.1)	1.000 *
Neutropenia	0 (0.0)	3 (6.1)	0.554 *

* Fisher’s exact test. † Mann–Whitney U test. Continuous variables are presented as mean ± standard deviation or median (interquartile range), as appropriate; categorical variables are presented as number (percentage). *p*-values were calculated using the independent-samples *t*-test, Mann–Whitney U test, Pearson’s chi-square test, or Fisher’s exact test, as appropriate. ECOG, Eastern Cooperative Oncology Group; IMDC, International Metastatic Renal Cell Carcinoma Database Consortium; IQR, interquartile range; SD, standard deviation; TKI, tyrosine kinase inhibitor.

**Table 2:** Multivariable Cox proportional hazards regression analysis of progression-free and overall survival.

Variable	PFS HR (95% CI)	*p*	OS HR (95% CI)	*p*
**Treatment: axitinib vs. nivolumab**	3.494 (1.690–7.225)	0.001	2.410 (1.200–4.838)	0.013
**IMDC risk group**	—	0.003 †	—	0.477 †
Intermediate vs. favorable	3.146 (1.570–6.302)	0.001	1.703 (0.664–4.366)	0.268
Poor vs. favorable	1.533 (0.467–5.035)	0.482	1.035 (0.544–1.971)	0.915
**Year of second-line treatment initiation**	1.070 (0.883–1.296)	0.489	1.102 (0.922–1.317)	0.286

HR, hazard ratio; CI, confidence interval; IMDC, International Metastatic Renal Cell Carcinoma Database Consortium. † Overall *p*-value for IMDC risk group.

## Data Availability

The data supporting the findings of this study are included within the article. Due to patient confidentiality and institutional regulations, the raw datasets are not publicly available.
